# Effect of temperature on in vitro germination and growth of *Colletotrichum fioriniae*, a new emerging pathogen of olive fruits

**DOI:** 10.1111/1758-2229.13275

**Published:** 2024-09-04

**Authors:** Federico Brugneti, Luca Rossini, Mounira Inas Drais, Silvia Turco, Angelo Mazzaglia

**Affiliations:** ^1^ Dipartimento di Scienze Agrarie e Forestali Università degli Studi della Tuscia Viterbo Italy; ^2^ Service d'Automatique et d'Analyse des Systèmes Université Libre de Bruxelles Brussels Belgium

## Abstract

Olive anthracnose induced by different *Colletotrichum* species causes dramatic losses of fruit yield and oil quality. The increasing incidence of *Colletotrichum fioriniae* (*Colletotrichum acutatum* species complex) as causal agent of olive anthracnose in Italy, is endorsing new studies on its biology, ecology, and environmental factors such as temperature. Five isolates from different sampling sites in Lazio region (Central Italy) were studied under controlled laboratory conditions aiming to better understand the differences of thermal development among the isolates and to lay the foundations of a future mathematical model able to describe the key aspects of the pathogen's life cycle. The mycelial growth rate and the conidial germination rate were assessed at seven different constant temperatures (5, 10, 15, 20, 25, 30, and 35°C) and fixed relative humidity (100% RH). The obtained dataset was analysed to estimate the parameters of mathematical functions that connect the mycelial growth rate and the spore germination with the environmental temperature. The parameters set provided as the result of this study constitute a key step forward in the biological knowledge of the species and the basis for future formulations of mathematical models that might be the core of decision support systems in an integrated pest management framework.

## Graphical abstract

The mycelial growth rate and the conidial germination rate of five isolates of *Colletotrichum fioriniae* were studied under seven different constant temperatures (5, 10, 15, 20, 25, 30, and 35°C) and fixed relative humidity (100% RH). The dataset was first analysed to assess differences among the isolates, and then was interpolated by mathematical functions that connect the mycelial growth rate and the spore germination with the environmental temperature.

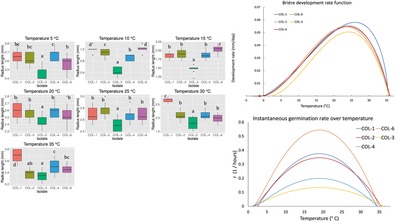

## INTRODUCTION

*Olea europaea* L. is among the most important tree crops worldwide, with a surface of over 12 million hectares devoted to the production of oil and table olives (https://www.internationaloliveoil.org). The greatest part of the production is concentrated in the Mediterranean basin, its native area, where Spain, Italy, Greece, Tunisia, and Portugal are the main growers (Fraga et al., 2020; Rossini, Bruzzone, et al., 2022). The olive plant is known for its excellent adaptability to different environmental and climatic conditions (Fraga et al., 2020). This feature endorsed the expansion of olive farming in Countries outside the native area, as recently occurred in different areas of south‐western Asia, Oceania, South Africa, and the Americas (Mousavi et al., 2019).

This phenomenon is gradually exposing olive plants to new abiotic and biotic stresses that affect the production. Moreover, the expansion of the olive cultivation and the climate change is also endorsing the re‐emergence of autochthonous diseases that are quickly expanding in areas where historically the outbreaks were contained. It is the case of the recent spread of the olive anthracnose, caused by different fungal species belonging to the genus *Colletotrichum*, which is leading to severe yield losses and to a strong decrease of the oil quality (Moral et al., 2021).

The species belonging to this genus have different biological behaviours, ranging from endophytic to necrotrophic, and are characterized by a high phenotypic and genotypic plasticity and diversity that explain its variability and aggressiveness (Cacciola et al., 2012; Moral et al., 2010, p. 20; Talhinhas et al., 2011; Talhinhas & Baroncelli, 2021). The ability of this group of fungi to survive and multiply without causing visible symptoms and/or remain in a quiescent state may explain why many producers suffer unexpected pre‐harvest losses by not observing symptoms during the season (Moral et al., 2021). Typical olive anthracnose symptoms consist of depressed, round, and ochre/brown lesions leading to fruit rot with evident great orange conidial masses. The pathogen can also cause the blight of olive flowers, mainly when mummies remain attached to the plant until the blossoming (Cacciola et al., 2012; Moral & Trapero, 2012), or cause the dieback of the branches because of the production of Aspergillomarasmine‐A, a phytotoxin produced in the rotten fruits (Ballio et al., 1969; Moral et al., 2021). The infected fruits mummify and fall on the soil in late autumn or in winter, under low temperature and higher relative humidity (RH) conditions.

To date, 18 *Colletotrichum* species have been associated with olive anthracnose worldwide (Garcia‐Lopez et al., 2023). These species belong to three *Colletotrichum* complexes, namely *Colletotrichum acutatum*, *Colletotrichum boninense*, and *Colletotrichum gloeosporioides*, but the most recent studies highlighted that the main species responsible of the disease are *C*. *acutatum* (sensu stricto), *Colletotrichum fioriniae*, *Colletotrichum godetiae*, *Colletotrichum nymphaeae*, *Colletotrichum rhombiforme*, and *Colletotrichum simmondsii* (Garcia‐Lopez et al., 2023; Moral et al., 2021). It is worth saying that multiple species can be present at the same time in the same olive growing areas, and in this case, it is typical to find a dominant and a secondary species (Garcia‐Lopez et al., 2023).

Surveys carried out along the Italian peninsula showed that, depending on the area, it is possible to find *C*. *godetiae*, *Colletotrichum aenigma*, *C*. *gloeosporioides*, *Colletotrichum cigarro* (*C*. *gloeosporioides* species complex), *Colletotrichum karstii*, *C*. *acutatum* (Faedda et al., 2011; Moral et al., 2021; Mosca et al., 2014; Schena et al., 2017), and, more recently, *C*. *fioriniae* (Riolo & Cacciola, 2022). Despite its first published report in 2023, according to the sequences deposited on the NCBI Genbank database, the association of *C*. *fioriniae* with olive tree can be traced back as early as 2014 (isolate 6MC, Accession numbers N° KX529647; MH547629; KX757972; KX757958; KX757935) in Northern Italy, in Central Italy in 2020 (isolates CREADC‐ER2203, CREADC‐ER2204, and CREADC‐ER2205, Accession numbers N° MT409123‐24‐26; MT379540‐41‐43) and in Viterbo province in 2022 (isolates COL‐1, COL‐2, COL‐3, COL‐4, and COL‐6 used in this study). In such a sense, monitoring carried out in the last years point to a significant increase of *C*. *fioriniae* incidence suggesting its primary role in olive anthracnose in Central Italy (personal observations from ongoing studies).

To date, the knowledge on key biological features of *C*. *fioriniae*, such as the thermic requirements for the mycelial growth and conidial germination, is still scarce. This lack of information hampers the formulation of proper control strategies and the development of decision support systems (DSS) based on mathematical models. DSS are gaining more importance in agriculture because of their capability to simulate different scenarios of plant infestations by insect pests or infections by fungal and bacterial pathogens (Capalbo et al., 2017; Knight & Mumford, 1994; Körner et al., 2014; Robinet et al., 2012). Having a reliable DSS would be a valuable tool to prevent or contain the spread of the diseases (Rossi et al., 2019). The first step toward modelling is to analyse, individually, the response of the pathogen to the external environment by accurate laboratory trials, where isolates of the fungus are cultivated under different constant conditions of temperature, humidity, and water activity.

This work aims to investigate the thermal response of different isolates of *C*. *fioriniae* collected in an important olive productive area of Central Italy, providing: (i) a detailed analysis, for the different isolates, of the mycelial growth under constant temperature conditions in a laboratory environment, and (ii) a first mathematical interpretation of the life cycle of this relevant pathogen, in particular of the mycelial growth and conidia germination rates. We believe that this set of quantitative information is a key propaedeutic step to extend the biological knowledge and to reach a final effective DSS for this pathogen. Laboratory experiments are, in fact, the first important step to carry out to provide the set of parameters that can be further implemented in more extended models, which, once developed, hold the potential for practical application in open field scenarios. Furthermore, the coexistence of various isolates within a single field highlights the importance of conducting this analysis, given that different responses to temperature fluctuations can be a synonym of shorter or longer risk of infection in function of the field microclimate conditions.

## EXPERIMENTAL PROCEDURES

### Fungal isolation, morphological, and molecular characterization

During the growing season 2022, a survey was carried out in 10 fields located in the Viterbo province (Lazio, Central Italy), in which 210 olive fruits showing typical anthracnose symptoms were collected and further analysed in the laboratory. The external surface of the collected olive fruits was sterilized in a 2% sodium hypochlorite solution for 2 min, and subsequently rinsed three times with sterilized distilled water. The olive fruits were then sectioned in slices of 1 mm of thickness and each slice was subsequently placed in a Petri dish with Potato Dextrose Agar (PDA). The Petri dishes were incubated at 25°C and 100% RH and inspected after 7 and 14 days. Colonies showing morphological traits of *Colletotrichum* spp. were placed in separate dishes to obtain pure cultures.

Genomic DNA from five representative isolates (COL‐1, COL‐2, COL‐3, COL‐4, and COL‐6) was extracted from 100 mg of fresh mycelium using the NucleoSpin Plant II mini kit (Macherey Nagel GmbH, Germany). Molecular characterization was carried out by Sanger sequencing of six partial genes (5.8S nuclear ribosomal gene with two flanking internal transcribed spacers [ITS], beta‐tubulin [TUB2], actin [ACT], partial sequences of the chitin synthase 1 [CHS‐1], histone 3 [HIS3], and a 200‐bp intron of the glyceraldehyde‐3‐phosphate dehydrogenase [GAPDH]). The primers used for the molecular identification (listed in the Appendix A) were ITS1 and ITS4, T1 Bt‐2b, ACT‐512F and ACT‐783R, CHS‐354R and CHS‐79F, CYLH3F and CYLH3R, GDF1, and GDR1, respectively.

### Phylogenetic relationship

The sequences obtained were concatenated and compared with the corresponding sequences from *C*. *fioriniae* and related species from NCBI, listed in Table S1. The latter include *C*. *nymphaeae*, *C*. *simmonsdii*, and *C*. *acutatum* as part of the *Colletotrichum acutatum* species complex, and the sequences of the isolates defined by Chen et al. (2022) and Zhang et al. (2023a) as *Colletotrichum orientalis* and *Colletotrichum radermacherae*, but deposited as *Colletotrichum* sp. or as *C*. *fioriniae*. The sequences of ITS, TUB2, ACT, CHS‐1, and GAPDH were concatenated and aligned to the sequenced amplicons of the five isolates from olive using MUSCLE v3.8.*31* (Edgar, 2004). The alignment file was the input for building a maximum likelihood (ML) phylogenetic tree using RAxML‐HPC v8.2.12 (Stamatakis, 2014), set with GTRCATI algorithm as a substitution model and 1000 bootstraps. The tree was visualized using FigTree v1.4.4 (http://tree.bio.ed.ac.uk/software/figtree/) and further edited with Inkscape v 0.92 (https://inkscape.org).

### Experimental design for mycelial growth at different constant temperatures

The mycelial growth rate of the selected *C*. *fioriniae* isolates was explored at seven constant temperatures, that is, 5, 10, 15, 20, 25, 30, 35°C, and fixed relative humidity of 100% RH, so that we could focus as much as possible on a single, and important, growth factor. We evaluated four replicates per isolate per each constant temperature to avoid errors due to potential anomalies in the growth, and the number of repetitions has been increased by measuring the radius in multiple directions, as detailed below in this section.

A 4 mm ∅ mycelial plug from 7 days‐old actively growing colony margins of each *Colletotrichum* isolate were placed on the centre of PDA medium (Microbiol®, Italy) plates. The plates were subsequently transferred in an incubator where temperature and humidity were maintained constant for all the duration of the experimentation.

**FIGURE 1:**
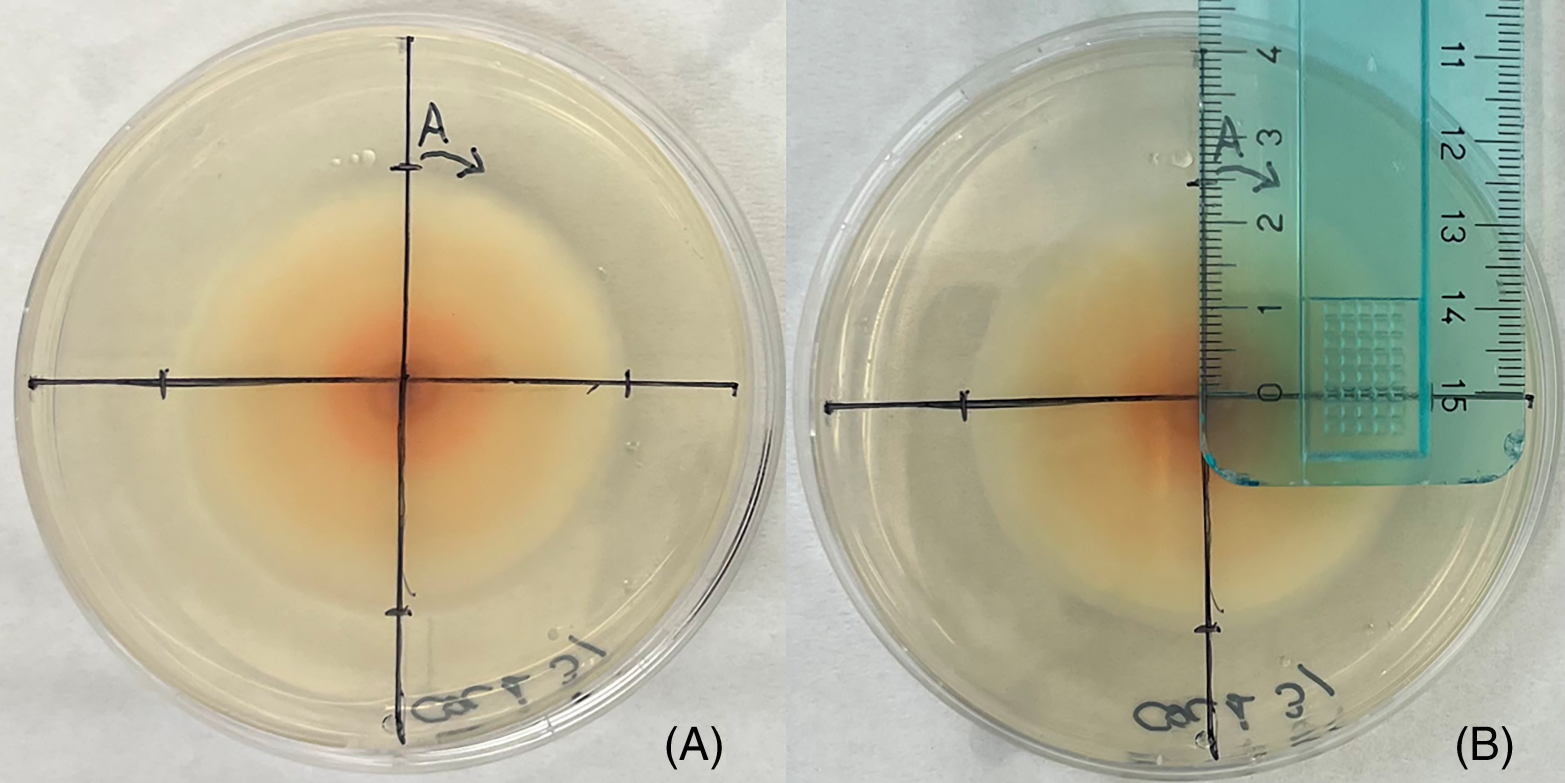
Measurement of mycelial growth. After 7 days the length of the radius was marked on the orthogonal axis (A) and subsequently measured with a ruler (±1 mm) (B). The example in the picture is specific for the isolate COL‐1 at 25°C.

The mycelial growth was measured with a ruler (±1 mm) after 7 days, a time range low enough to ensure that the fungus did not reach the border of the dishes, following the four orthogonal directions (N‐W‐S‐E) from the centre of the plug (Figure 1). The four directions measured in each dish ulteriorly increase the precision of the measure of the radius.

### Experimental design for conidial germination at different constant temperatures

Conidia were scraped from the mycelium and subsequently filtered using four layers of cheesecloth to remove any bigger fragment (Drais et al., 2021). The conidial suspension was then quantified using a hemo‐cytometer and diluted to obtain a final concentration of 10^5^ spore/mL. After the quantification, 5 μL of conidial suspension was placed on 4 mm ∅ Water Agar (Concentration of 12 g/L) plugs placed on a microscope glass slide. The slides were subsequently placed on Petri dishes and incubated at constant temperature conditions of 5, 10, 15, 20, 25, 30, and 35°C and 100% of relative humidity, analogously to the mycelial growth. Four replicates per isolate per each constant temperature were evaluated.

**FIGURE 2:**
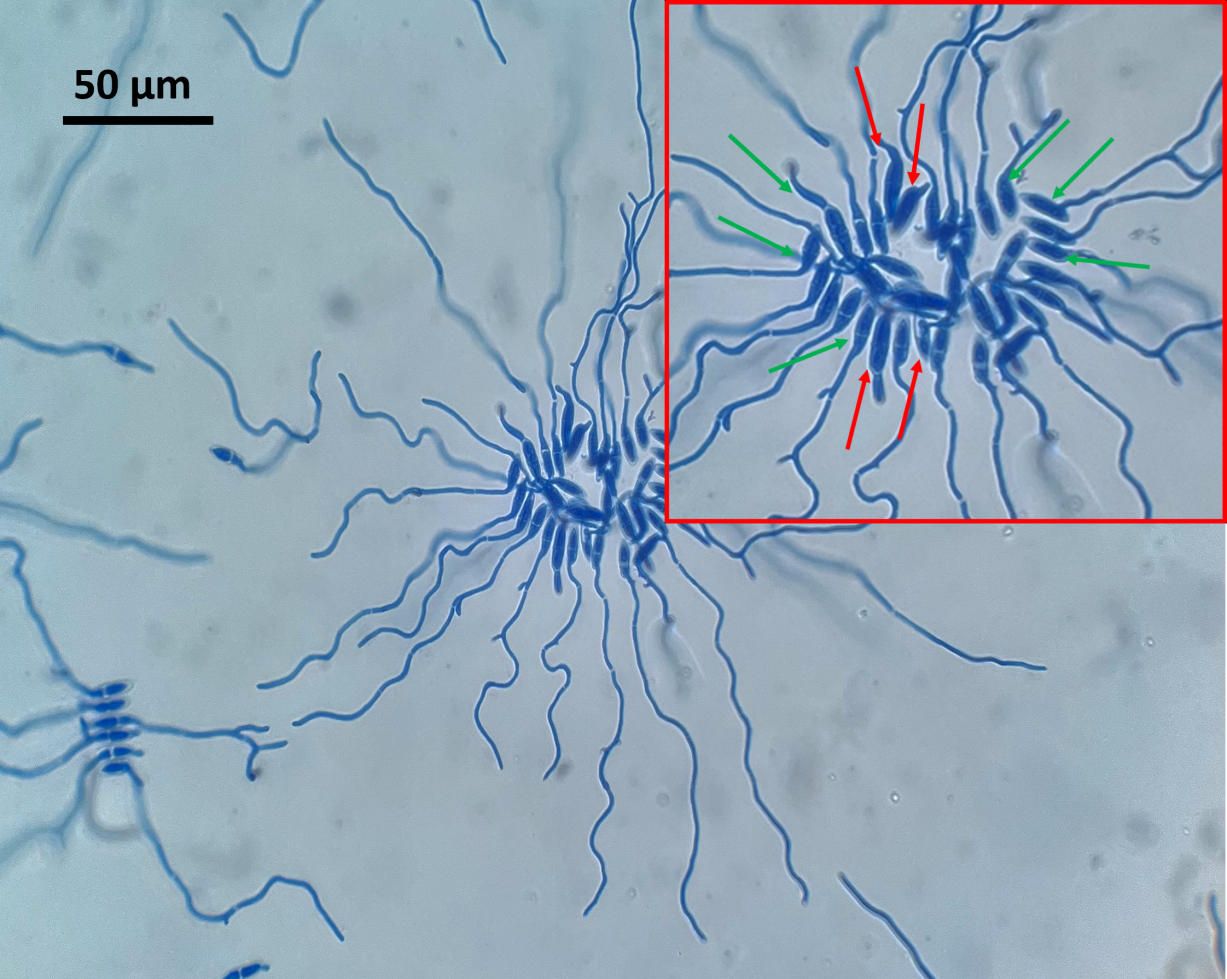
Count of the germinated and non‐germinated conidia after 15 h at 25°C. Conidia were considered as germinated once the length of the germ tube was more than one half of the length of the spore, as indicated by the green arrows. Red arrows indicate examples of non‐germinated conidia. Measurements were carried out at 40× magnification.

The conidia were considered as ‘germinated’ once the length of the germ tube was more than one half of the length of the spore, as shown in Figure 2. Germinated conidia were counted at fixed time ranges of 6, 10, 15, 20, 24, and 48 h by staining with lactophenol cotton blue. The percentage of germinated spores was determined by observing random groups of 100 conidia per replicate, so that the results are directly expressed as a percentage.

### Mycelial growth rate data analysis

Before the modelling approach, the mycelial growth dataset was analysed in a more classical way using the RStudio software v. 4.3.2 (R Core Team, 2018), so that we could highlight eventual differences among the isolates. Notably, we first carried out a wider analysis of the whole dataset, then we focused on the response of the isolates to each constant temperature explored in the experimentation.

#### Whole dataset

Before the analysis, we first checked the normality of the complete dataset through a Shapiro–Wilk test, using the *shapiro*.*test*() function within the basic R environment, and through a visual inspection of the Quantile–Quantile (Q‐Q) plot, draw using the *qqmath*() function within the *lattice* R package (Sarkar, 2008). Given the non‐normal trend of the dataset, the best function to transform the dataset was chosen by using the *bestNormalize*() function within the R package *bestNormalize* (Peterson, 2021; Peterson & Cavanaugh, 2020). The transformed dataset was then analysed through a linear model (LM) by using the *lmer*() function within the R package *lme4* (Bates et al., 2015) and considering temperature and isolate as independent variables and the plate and the orthogonal direction (N‐W‐S‐E) of the measured radii as random effects. The LM was subsequently followed by a Bonferroni post hoc test (α = 0.05) to assess the differences among the temperatures and the isolates by using: the *emmeans*() function within the R package *emmeans* (Searle et al., 1980), the *pairs*() function within the R package *multicompView*, and the *cld*() function within the R package *multcomp* (Hothorn et al., 2008).

#### Temperature‐by‐temperature analysis

Before this second part, the whole dataset was divided into specific sub‐datasets, where the measures of the radii of the different isolates were grouped by temperature. The normality trend of each sub‐dataset was checked before the analysis, as well as eventual transformations were carried out in the same way of the general dataset. The analysis procedure was the same as the general dataset, except for the use of a LM considering only the isolate as independent variable and plate and orthogonal direction of the measured radii as random effect.

### Modelling the mycelial growth rate in function of temperature

A single value of the radius from each replicate was obtained from the dataset of each isolate by considering the average and the standard deviation of the four radial distances measured after 7 days. The average radius of each Petri dish was initially expressed in millimetres per week (mm/w), but a further conversion was carried out by dividing the mean and the associated standard deviation by 7, so that the unit was mm/day (Drais et al., 2023). The converted dataset of each isolate was subsequently interpolated with the Briére equation (Briere et al., 1999), whose mathematical expression is the following:

(1) $$R\left[T\right]=a\;T\;\left(T-T_{L}\right){\left(T_{M}-T\right)}^{1/m},$$

where T is the temperature of growth, a and m are empirical parameters, and $T_{L}$ and $T_{M}$ are the minimum and maximum thresholds for the mycelial growth, respectively. The parameters and the associated standard errors of the Equation (1) were obtained by least squares fit, while the goodness of fit was evaluated through a $\chi^{2}$‐test and the coefficient of determination $R^{2}$ (Bellocchi et al., 2011; Ikemoto & Kiritani, 2019; Rossini et al., 2019a, 2019b; Rossini, Bono Rosselló, et al., 2021; Rossini, Severini, et al., 2020; Rossini, Speranza, & Contarini, 2020; Rossini, Virla, et al., 2021).

The Equation (1) has a typical increasing‐decreasing profile with a maximum, coinciding with the optimal temperature, $T_{\mathrm{opt}}$, for mycelial growth. By placing the first derivative of temperature to zero, namely $\frac{d}{\mathrm{dT}}R\left[T\right]=0$, it is possible to obtain the abscissa of the maximum (Briere et al., 1999; Rossini, Bono Rosselló, et al., 2022; Rossini, Bruzzone, et al., 2022; Rossini, Severini, et al., 2020):

(2) $$T_{\mathrm{opt}}=\frac{2mT_{M}+T_{L}\left(m+1\right)+\sqrt{4m^{2}T_{M}^{2}+T_{L}^{2}{\left(m+1\right)}^{2}-4m^{2}T_{M}T_{L}}}{4m+2},$$

additional quantitative information of fundamental importance. It is worth remarking that the least squares fit provides the errors associated with the parameters, so that it is possible to calculate the error associated with $T_{\mathrm{opt}}$ by applying the propagation of the uncertainty formula to the Equation (2), as detailed in Rossini, Contarini, et al. (2020).

### Modelling the conidial germination rate

The experimental setup to assess the conidial germination under different temperatures provided a dataset distributed according to a logistic function. The main assumption is that as time passes, the percentage of germinated conidia reaches 100%, more or less faster depending on temperature. The dataset of each isolate at each constant temperature listed in Experimental design for mycelial growth at different constant temperatures section was interpolated with the following logistic equation describing the germination rate over time (Gabriel y Galán et al., 2015; Prosser, 1995):

(3) $$G\left(t\right)=\frac{kG_{0}}{G_{0}+\left(k-G_{0}\right)e^{-\mathrm{rt}}}.$$

The parameters k, $G_{0}$, and r in Equation (3) are respectively the carrying capacity, the number of germinated conidia after 6 h (the moment of the first sampling), and the instantaneous germination rate. The experimental set up led us to consider k=100, with the advantage of estimating only two parameters (*G*_0_ and *r*) instead of three. As for the Briére equation (Experimental design for conidial germination at different constant temperatures section), the parameters were estimated through a non‐linear least squares regression, while the goodness of fit was evaluated through a *χ* ^2^‐test and considering the coefficient of determination $R^{2}$. Before the fitting operation, the dataset of each isolate and for each constant temperature was organized considering the four values measured at each sampling time, for a total of 24 values.

From the step described above we obtained the profiles of $G_{0}$ and r over temperature. We can therefore interpolate the instantaneous germination rate over temperature, T, using the following equation (Gougouli & Koutsoumanis, 2012; Omuse et al., 2021; Rosso et al., 1993, 1995):

(4) $$r\left[T\right]=\frac{r^{\mathrm{opt}}\left(T-T_{r}^{\max}\right){\left(T-T_{r}^{\min}\right)}^{2}}{\left(T_{r}^{\mathrm{opt}}-T_{r}^{\min}\right)\left[\left(T_{r}^{\mathrm{opt}}-T_{r}^{\min}\right)\left(T-T_{r}^{\mathrm{opt}}\right)-\left(T_{r}^{\mathrm{opt}}-T_{r}^{\max}\right)\left(T_{r}^{\mathrm{opt}}+T_{r}^{\min}-2T\right)\right]}$$

where $r^{\mathrm{opt}}$, $T_{r}^{\max}$, $T_{r}^{\min}$, $T_{r}^{\mathrm{opt}}$ represent the value of the instantaneous germination rate at the optimal temperature and the maximum, minimum, and optimal temperature for the conidial germination, respectively. The interpolation was carried out through a non‐linear fit using the least squares method, but given the reduced number of degrees of freedom, the goodness of fit has been entrusted only on the coefficient of determination $R^{2}$.

### Parameters estimation and analysis of the Equations (1–4)

The non‐linear regressions mentioned in Mycelial growth rate data analysis and Modelling the mycelial growth rate in function of temperature sections were carried out through ad hoc Python scripts publicly available at https://github.com/lucaros1190/Colletotrichum‐Temperature. The repository also contains the raw dataset, the R script used in Experimental design for conidial germination at different constant temperatures section, and all the additional information to fully reproduce the results of this study.

## RESULTS

### *Morphological characterization of* C. fioriniae *isolates*

**FIGURE 3:**
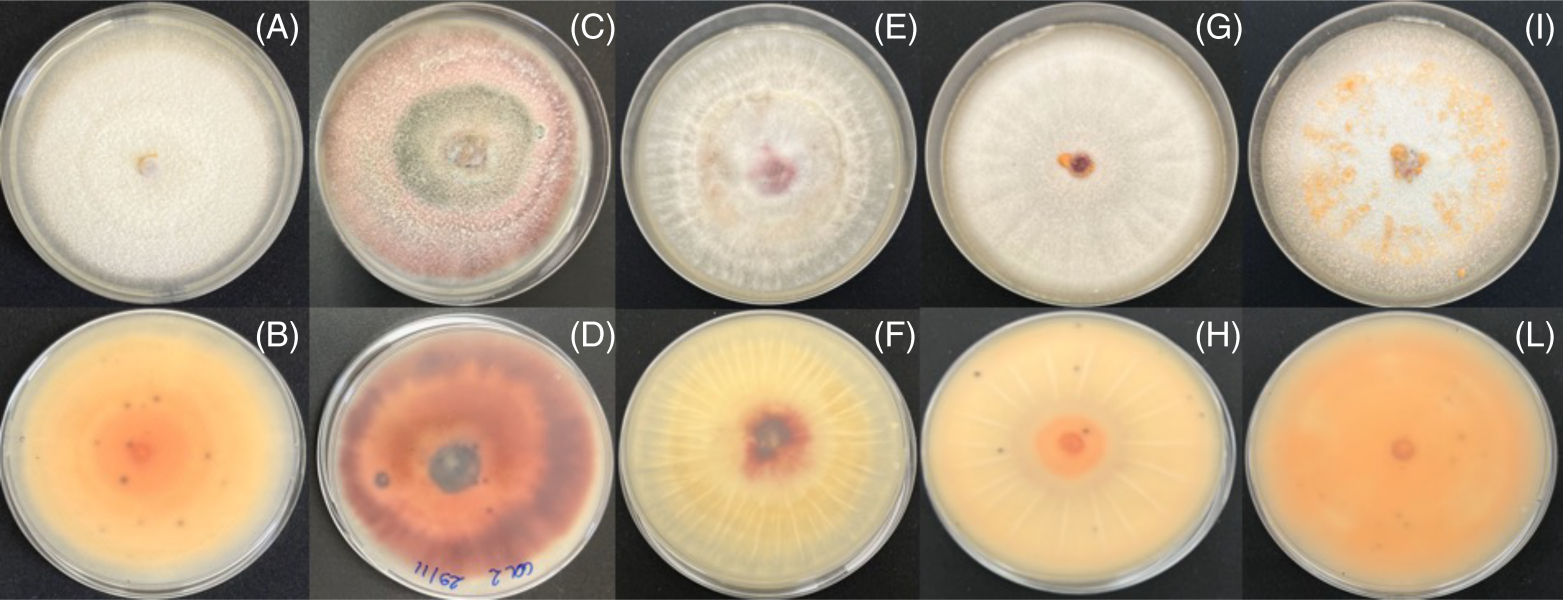
Morphology of the colonies on PDA substrate after 10 days at 25°C of the isolates used in this study. For each isolate is reported the upper and reverse side of the plate: (A,B) COL‐1; (C,D) COL‐2; (E,F) COL‐3; (G,H) COL‐4; (I,L) COL‐6.

Five representative isolates were chosen for downstream analysis, according to the uniqueness of their morphological traits. After 10 days at 25°C, pure cultures of the five isolates showed a grey to orange cottony and aerial mycelium, from pale orange to dark red in reverse (Figure 3). Conidia were hyaline, smooth‐walled, aseptate, narrowly elliptical pointed at both ends, measuring 11–21 μm (mean 15.5 μm) × 3.5–7 μm (mean 4.5 μm), and contained in orange masses.

### *Molecular and phylogenetic characterization of* C. fioriniae *isolates*

After PCR amplification of the six partial genes and Sanger sequencing, the BLASTn analysis revealed 100% identity of our isolates with the deposited sequences of *C*. *fioriniae*. Our sequences were deposited under the GenBank Accession no. ON773228‐ON773232 for ITS, ON807567‐ON807571 for TUB2; ON791496‐ON791500 for ACT, ON791501‐ON791505 for CHS‐1, ON791506‐ON791510 for HIS3, ON791511‐ON791515 for GAPDH, respectively (Table 1).

**TABLE 1:** Isolates of *Colletotrichum fioriniae* characterized in this study.

Acronym	Location of the olive orchard and year	GenBank accession number					
		ITS	TUB2	ACT	CHS‐1	HIS3	GAPDH
COL‐1	Viterbo (IT), 2022	ON773228.1	ON807567.1	ON791496.1	ON791501.1	ON791506.1	ON791511.1
COL‐2	Caprarola (IT), 2022	ON773229.1	ON807568.1	ON791497.1	ON791502.1	ON791507.1	ON791512.1
COL‐3	Sutri (IT), 2022	ON773230.1	ON807569.1	ON791498.1	ON791503.1	ON791508.1	ON791513.1
COL‐4	Viterbo (IT), 2022	ON773231.1	ON807570.1	ON791499.1	ON791504.1	ON791509.1	ON791514.1
COL‐6	Viterbo (IT), 2022	ON773232.1	ON807571.1	ON791500.1	ON791505.1	ON791510.1	ON791515.1

The phylogeny inferred by ML analysis (Figure S1) indicates that the olive isolates under investigation are included in the *C*. *fioriniae* cluster. *Colletotrichum orchidophilum* CBS‐632.80 was here used as an outgroup.

### Mycelial growth rate—Experimental data analysis

From the first step of the analysis described in Mycelial growth rate data analysis section, we assessed that the mycelial growth of the overall isolates over temperature was significantly different (LM, *p* < 0.0001), namely there were significant differences among all the experimental temperatures explored. For the sake of exposition, we hereafter report only the *p*‐values obtained by the analysis in Mycelial growth rate data analysis section, referring the most interested reader to the shared scripts (Parameters estimation and analysis of the Equations (1–4) section) and dataset for further details. Additionally, in case of multiple comparisons, the greater *p*‐value is reported between parentheses after the results, so that the other ones are implicitly meant smaller.

**FIGURE 4:**
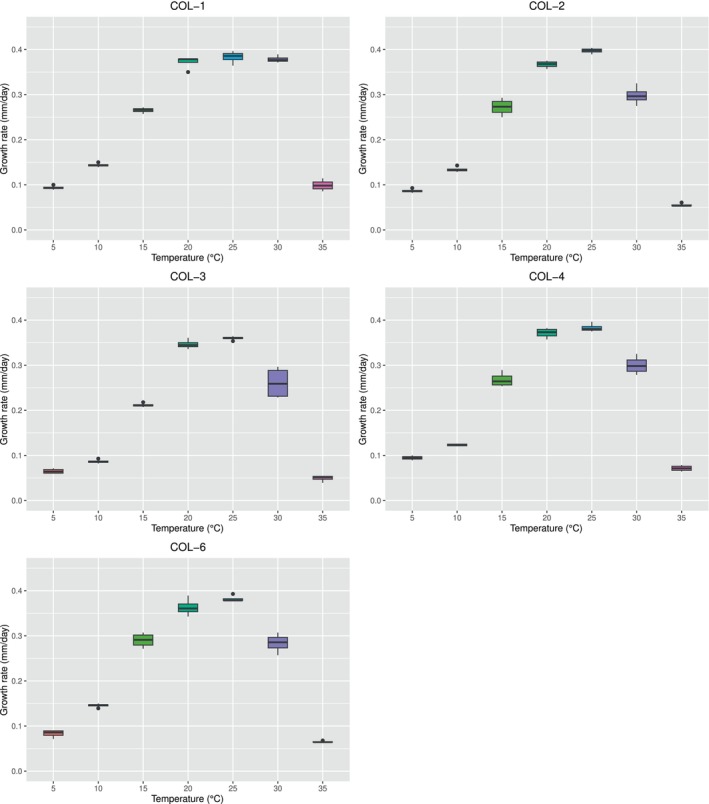
Mycelial extension in terms of radial growth (millimetres per day) at different constant temperatures (°C). Each graph reports a boxplot of the raw data corresponding to each isolate considered in this study.

An overall idea of the experimental dataset is provided by the boxplots in Figure 4, reporting the raw data (converted in mm/day) collected for each isolate. Notably, the dataset of all the isolates presents an increasing‐decreasing profile, with a well identifiable maximum coinciding with the optimal temperature for the mycelial growth. The absence of outliers suggests that there were no anomalies referable to systematic errors during the experimentation, even if they were considered in the LM analysis by the two random effects (plate and orthogonal direction).

Excluding temperature as a factor, it was assessed that the isolates COL‐2, COL‐4, and COL‐6 showed no overall statistical differences in the mycelial extension rate (LM, *p* = 1), while the isolates COL‐1 and COL‐3 were different to each other, and to the rest of the isolates (LM, *p* < 0.0001).

**FIGURE 5:**
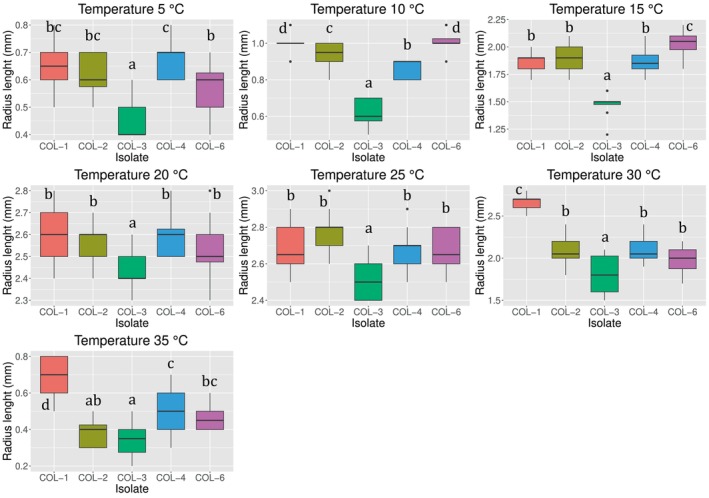
Mycelial extension in terms of radial growth (millimetres per week) at different constant temperatures (°C). Each graph reports a boxplot of the dataset grouped by temperatures. Different letters mean statistical differences assessed with a linear model followed by a Bonferroni post hoc test (*α* = 0.05).

By grouping the dataset by temperature, instead, it was possible to appreciate the differences among the isolates at the various temperatures with a major detail. As shown in Figure 5, at 5°C the isolates COL‐3, COL‐4, and COL‐6 were statistically different from each other (LM, *p* < 0.047), while the isolates COL‐1 and COL‐2 were statistically different only from COL‐3 (LM, *p* < 0.0001). At 10°C the isolates COL‐1 and COL‐6 did not report statistical differences (LM, *p* = 1) while being both different from the other isolates (LM, *p* < 0.0001); COL‐2, COL‐3, and COL‐4 were different from each other as well, (LM, *p* < 0.001). At 15°C the isolate COL‐3 and COL‐6 showed the lowest and the highest mycelial extension rate, respectively, statistically different from the other isolates (LM, *p* < 0.002); the isolates COL‐1, COL‐2, and COL‐4, instead did not show statistical differences (LM, *p* = 1). The growth at 20 and 25°C showed the same behaviour, with no statistical differences among the isolates COL‐1, COL‐2, COL‐4, and COL‐6 (LM, *p* = 1). The isolate COL‐3, instead, was the lowest one in terms of mycelial extension, and it was different from the other isolates (LM, *p* < 0.0001). At 30°C, COL‐1 and COL‐3 showed the highest and the lowest mycelial extension rate, respectively, and they were different both from each other and from the other isolates (LM, *p* < 0.001). Conversely, the isolates COL‐2, COL‐4, and COL‐6 were not different from each other (LM, *p* > 0.6). At 35°C, the last temperature explored, the isolates COL‐3 and COL‐1 were the lowest and the highest in terms of mycelial extension rate, respectively, and statistically different from each other (LM, *p* < 0.0001). The isolate COL‐3 was not statistically different from COL‐2 (LM, *p* = 1), as well as the isolates COL‐2 and COL‐6, and COL‐6 and COL‐4 (LM, *p* > 0.3). The only additional difference was observed between the isolates COL‐2 and COL‐4 (LM, *p* = 0.009).

In light of these results, it is noteworthy that both lower and upper temperatures provoke a differentiation among the isolates, highlighting their positive or negative response to temperature variations. Conversely, the lower differences occurred between 20 and 30°C, indicating that this range includes the optimal temperature for mycelial growth. This first analysis of the dataset highlights important aspects that will be further refined in the subsequent section, where we present the results of the model parameters' estimation.

### Mycelial growth rate—Mathematical interpretation

**FIGURE 6:**
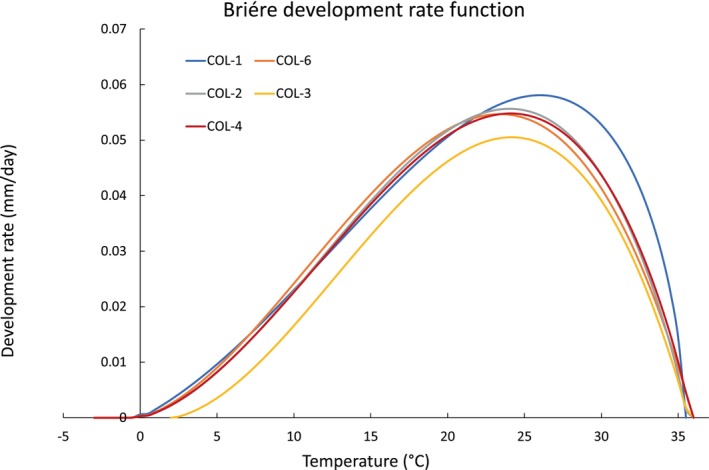
Best fit functions (1) describing the mycelial growth rate over temperature of each isolate considered in this study. The corresponding parameters and their standard errors are listed in Table 2.

According to the experimental and data analysis protocols described in Modelling the mycelial growth rate in function of temperature section, the first result concerns the measurement of the mycelial extension rates over temperature and the subsequent estimation of the parameters of the function (1). A quantitative interpretation of the dataset was provided by the interpolation of the dataset with the Briére Equation (1), whose results are listed in Table 2 and graphically represented in Figure 6. The overall optimal temperature for the mycelial growth is around 24°C, with a slightly lower value of $T_{\mathrm{opt}}=\left(23\pm 1\right)$°C calculated for the isolate COL‐6 and a slightly higher value of *T*_opt_ = (25 ± 4)°C for the isolate COL‐1. Besides the variations, the optimal temperatures for the mycelial growth are all in accordance with each other, considering the associated standard errors.

**TABLE 2:** Best fit parameters (±standard error) of the Briére function (1) describing the mycelial extension rate over temperature.

Isolate	Briére function's parameters	Goodness of fit and optimal temperature
COL‐1	$a=\left(1.07\pm 0.09\right)\cdot 10^{-6}$	$\chi^{2}=0.00257^{*}$
	$T_{L}=-10\pm 4$	$R^{2}=0.978$
	$T_{M}=35.4\pm 0.2$	NDF=24
	m=1.6±0.2	$T_{\mathrm{opt}}=25\pm 4$
COL‐2	$a=\left(6.4\pm 0.9\right)\cdot 10^{-5}$	$\chi^{2}<10^{-4*}$
	$T_{L}=-3\pm 2$	$R^{2}=0.979$
	$T_{M}=35.6\pm 0.2$	NDF=24
	m=1.09±0.08	$T_{\mathrm{opt}}=24\pm 1$
COL‐3	$a=\left(6\pm 1\right)\cdot 10^{-5}$	$\chi^{2}=0.00441^{*}$
	$T_{L}=2\pm 1$	$R^{2}=0.955$
	$T_{M}=35.7\pm 0.3$	NDF=24
	m=1.0±0.1	$T_{\mathrm{opt}}=24\pm 1$
COL‐4	$a=\left(6\pm 1\right)\cdot 10^{-5}$	$\chi^{2}=0.00403$
	$T_{L}=-3\pm 2$	$R^{2}=0.966$
	$T_{M}=35.8\pm 0.3$	NDF=24
	m=1.0±0.1	$T_{\mathrm{opt}}=24\pm 2$
COL‐6	$a=\left(4.7\pm 0.8\right)\cdot 10^{-5}$	$\chi^{2}=0.00011$
	$T_{L}=-3\pm 2$	$R^{2}=0.980$
	$T_{M}=36.0\pm 0.3$	NDF=24
	m=0.98±0.07	$T_{\mathrm{opt}}=23\pm 1$

*Note*: Additional information is reported by the coefficient of determination $R^{2}$, by the number of degrees of freedom (NDF), and $\chi^{2}$ value. Figure 3 shows the raw dataset utilized for parameter estimation, while Figure 4 provides a graphical representation of the best fit functions. Optimal temperature $T_{\mathrm{opt}}$ was calculated through Equation (2). The (*) symbol above the $\chi^{2}$ values mean a significance of *p* < 0.01 to the $\chi^{2}$‐test.

A higher variability among the isolates was observed in the minimum temperatures for the mycelial growth, $T_{L}$. The values, in fact, range from *T*_L_ = (−10 ± 4)°C calculated for COL‐1, to *T*_L_ = (2 ± 1)°C of COL‐3 while the other isolates are around a *T*_*L*_ value of −3°C. The maximum temperatures for the mycelial growth showed a lower variability, with values included between 35.4 and 36°C. These results are in line with the analysis of the dataset described in Mycelial growth rate data analysis section and provide a higher level of definition.

### Conidial germination rate

The data collected from this part of the experimentation allowed us to estimate the parameters of the logistic Equation (3), reported as a supplementary material with standard errors and goodness of fit values (see the repository link). As described in Modelling the conidial germination rate section, the Equation (3) provided the instantaneous and the initial germination rate over temperature for each of the isolates considered in this study.

**FIGURE 7:**
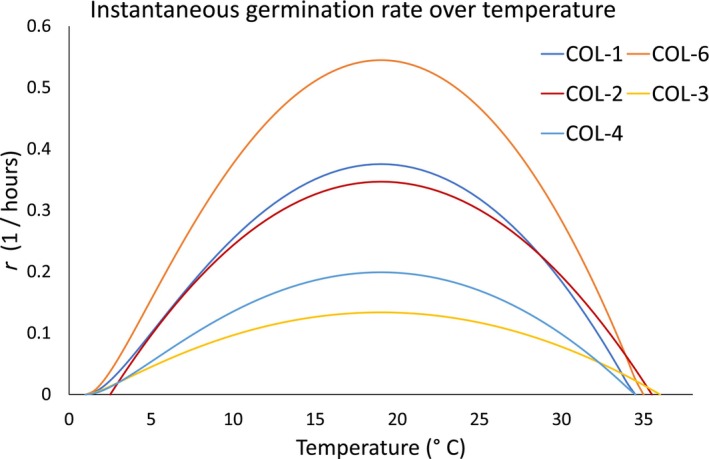
Best fit functions describing the instantaneous germination rate $r\left[T\right]$ of the isolates considered in this study, and mathematically described by the Equation (4). The best fit values and their standard errors are instead listed in Table 3.

The instantaneous germination rate over temperature was described by the function (4), whose parameters, specific for each isolate considered in this study, are listed in Table 3, and that are graphically represented in Figure 7. The optimal temperature for the germination, $T_{r}^{\mathrm{opt}}$, was the same (19°C) for all the isolates, in the limits of the standard errors. This result may indicate a similarity between the isolates, at least in terms of adaptation to the environment, however the main differences assessed can be found by looking at the values of the conidial germination rate at the optimal temperature, $r^{\mathrm{opt}}$, (Table 3). These values showed an overall variability, whose extremes are represented by the slower isolate COL‐3 ($r^{\mathrm{opt}}=0.13\pm 0.03$) and the faster one COL‐6 ($r^{\mathrm{opt}}=0.5\pm 0.1$). The minimum temperatures for the conidial germination were all concentrated between 1 and 2°C, with higher uncertainties, if compared with the other parameters. This is likely due to the fitting algorithm, which may have higher difficulties in fitting values next to zero. The upper limits for the conidial germination were concentrated among 34 and 36°C, with COL‐1 and COL‐4 the least and COL‐3 the most tolerant to higher temperatures.

**TABLE 3:** Best fit parameters (± standard errors) estimated for the function (4) describing the instantaneous germination rate over temperature, $r\left[T\right]$.

Isolate	Best fit parameters values	Goodness of fit parameters
COL‐1	$r^{\mathrm{opt}}=0.37\pm 0.06$	
	$T_{r}^{\max}=34\pm 2$	$R^{2}=0.802$
	$T_{r}^{\min}=1\pm 11$	NDF=3
	$T_{r}^{\mathrm{opt}}=19\pm 3$	
COL‐2	$r^{\mathrm{opt}}=0.35\pm 0.09$	
	$T_{r}^{\max}=35\pm 4$	$R^{2}=0.553$
	$T_{r}^{\min}=2\pm 2$	NDF=3
	$T_{r}^{\mathrm{opt}}=19\pm 4$	
COL‐3	$r^{\mathrm{opt}}=0.13\pm 0.03$	
	$T_{r}^{\max}=36\pm 3$	$R^{2}=0.582$
	$T_{r}^{\min}=1\pm 30$	NDF=3
	$T_{r}^{\mathrm{opt}}=19\pm 4$	
COL‐4	$r^{\mathrm{opt}}=0.20\pm 0.03$	
	$T_{r}^{\max}=34\pm 2$	$R^{2}=0.775$
	$T_{r}^{\min}=1\pm 12$	NDF=3
	$T_{r}^{\mathrm{opt}}=19\pm 3$	
COL‐6	$r^{\mathrm{opt}}=0.5\pm 0.1$	
	$T_{r}^{\max}=35\pm 3$	$R^{2}=0.601$
	$T_{r}^{\min}=1\pm 17$	NDF=3
	$T_{r}^{\mathrm{opt}}=19\pm 4$	

*Note*: Additional information is reported by the coefficient of determination $R^{2}$ and by the number of degrees of freedom (NDF).

According to the values listed in Table 3, COL‐3 is the isolate whose conidia germinate slower at the optimal temperature, but at the same time it covers a higher thermal spectrum, between 2 and 3°C wider with respect to the other isolates. Analogously, COL‐6 seems to be more tolerant to higher temperatures (just 1°C less than COL‐3), but the germination rate at the optimal temperature is the highest one, suggesting a prompt response in starting the infection on new plants.

**FIGURE 8:**
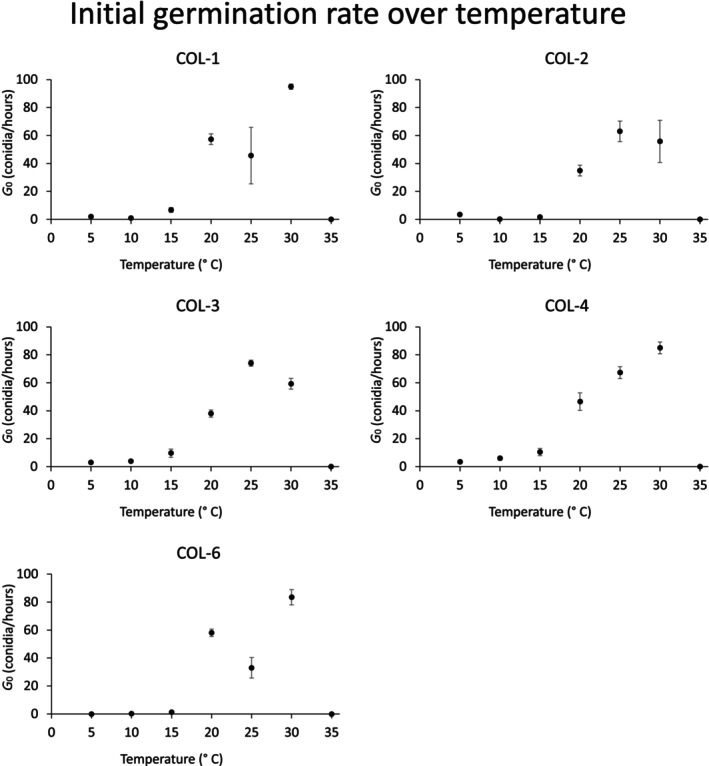
Initial germination rate $G_{0}$ over temperature for the isolates considered in this study.

The initial germination rates over temperature, instead, are graphically represented in Figure 8. Even if there is no further interpolation of these data, as we did for the instantaneous germination rates $r\left[T\right]$, an analysis of the percentage of germinated conidia after 6 h provides an idea of the timing for an eventual control action for given environmental conditions. As observable in Figure 8, there are different scenarii among the isolates: for instance, COL‐2 and COL‐3 seem to have an increasing‐decreasing profile where at 25°C the 63% and 74% of the conidia are already germinated, respectively. On the other hand, COL‐1, COL‐4, and COL‐6 reach percentages of 95%, 85%, and 83% of the conidia germinated at 30°C, suggesting a higher potential of infection at higher temperatures.

## DISCUSSION AND CONCLUSION

*Colletotrichum fioriniae* is a widespread fungal species known for causing anthracnose in several crops. Belonging to the *C*. *acutatum* species complex, it was initially recognized as *C*. *acutatum* var. *fioriniae* (Marcelino et al., 2008) and the year after upgraded to *C*. *fioriniae* as a separated species by Shivas and Tan (2009). In a more recent paper, two subclades of *C*. *fioriniae* were proposed as separated species, namely *C*. *fioriniae* and *C*. *orientalis* (Chen et al., 2022), as well as a third group of closely related isolates were assigned to the new species *C*. *radermacherae* (Zhang et al., 2023a). In our phylogenetic analysis, the isolates are clustered both with *C*. *fioriniae* and *C*. *orientalis* (indicated as *Colletotrichum* sp. as deposited on the NCBI GenBank database). However, the very few nucleotide polymorphisms existing along the entire concatenated 5 gene sequences (7 SNPs out of 1600 bp) of these isolates raise uncertainties about their taxonomic arrangement in different species instead of being considered as representatives of intraspecific genetic variability, as originally proposed by Damm et al. (2012) and recently assessed by Zhang et al., 2023b. Such questions are not extraordinary in fungal taxonomy (Hilário et al., 2021) and they have already interested other species complexes of the *Colletotrichum* genus, requiring specific coalescent approaches to be solved (Liu et al., 2016). With this premise, all the five isolates characterized in this study were classified, and their sequences were deposited, as *C*. *fioriniae*.

Here, we provided a set of information on the thermal response of the mycelial growth and the conidial germination rate of these five isolates, together with a set of biological parameters included in mathematical functions that provide a more refined description of the results obtained by laboratory experiment. Given the entity of the damages that this pathogen is causing on olive cultivations, a mathematical interpretation of its life cycle with respect to the effect of the external environment is of great help. This study is propaedeutic to further develop specific DSS that may be of great help to predict and effectively constrain the incidence of the olive anthracnose and will be fundamental for planning strategic field interventions to control this pathogen. Targeted interventions when environmental conditions are favourable to the development of the pathogen can lead to a significant reduction in disease incidence, a better management of costs, and an optimization of the use of plant protection products. It is worth remarking that we explored only the dependence on temperature, neglecting the role of the water *lato* sensu. While we recognize that parameters such as relative humidity, rain, leaf wetness or dew are important for the development of fungi, we are deferring these aspects to future studies, in order to concentrate our conclusion on individual parameters. Conversely, the choice for this work was the comparison of the thermal response of different isolates that coexist within the same olive productive area, an interesting aspect from a phytopathological point of view. As outlined in Introduction section, the prevalence of multiple isolates, even on the same plant, is a common occurrence for this pathogenic agent (Garcia‐Lopez et al., 2023), and only simple and well‐focused laboratory experiments can provide clearer indications about their coexistence and interaction.

Our results showed different scenarii for the *Colletotrichum* isolates explored, presenting an overall variability of the parameters that may correspond to an earlier or delayed infection depending on the environmental conditions. Moreover, the differences assessed among the isolates, above all to low and high temperatures, leave us to suppose that the primary and secondary isolate responsible of olive anthracnose may change from field to field depending on the microclimate variations. In most of the cases, *Colletotrichum* spp. is present in the environment in crop residues and in mummified fruits, the main source of inoculum for the following season (Cacciola et al., 2012). The primary inoculum is caused by fresh conidia produced in acervuli at the beginning of the growing season as soon as the environmental conditions are favourable. According to the existing literature, there is production and germination of conidia once relative humidity values overcome 95% and temperature ranges between 10 and 30°C, while the formation of the appressoria reaches maximum levels as the RH approaches 100% (Estrada et al., 2000; Moral & Trapero, 2012). Our study quantitatively completes this information, providing a profile of the instantaneous germination rate at different constant temperatures. It is worth noting however that while the Briére Equation (1) correctly described the mycelial growth rates over temperature, the values related to the minimum thresholds $T_{L}$ should be regarded more as a theoretical limit. The reason is behind the mathematical structure of the Briére equation, that makes the least square fitting operation challenging for this specific parameter (Jin et al., 2022). A consequence is the higher relative error associated to $T_{L}$, if compared with the other ones. In contrast to the Briére (1), the Rosso Equation (4) had a lower performance in terms of data fitting. The reason can be the high variability of the dataset of the conidial germination, probably due to the fast germination that occurs at temperatures near the optimum. The reactivity of the isolates in conidial germination was quantitatively described by the parameter $G_{0}$ of the logistic function (3), that clearly shows the different behaviour. At optimal temperatures, the overall isolates showed high germination rates after just 6 h, with differences mostly concentrated on high temperatures more than lower ones. As a possible consequence, we may say that while the germination of the conidia during the spring and autumn is more or less similar, during the summer, when warmer temperatures happen, there are some isolates more performant than others, representing a higher risk for the ripening fruits.

Considering the minimum temperature values for the germination, we can say that the conidia present in the environment can germinate, in Central Italy, even at the end of the winter, when the level of relative humidity and water availability in sensu *latu* is often high. If we consider, in addition, the temperature‐dependent mycelial growth rate, we can conclude that after the germination, the mycelium is capable of growing at the same conditions, even if slowly.

The considerations discussed in the previous paragraphs are in line with the existing literature and justify why the symptoms become more visible as the fruit ripening is in an advancing state. Early in the season, the conidial germination and the subsequent mycelial growth are gradual, reaching a peak in late May. This fact is justified by our results, given that the optimal temperature is of ~19 and 23°C for conidial germination and mycelial growth, respectively. These temperatures, on average, are prevalent during mid‐spring and autumn. While the higher summer temperatures may slow down the development of the fungus, it can anyway reach and affect the small olives. At this point, the dry condition of the summer stops the growth of the pathogen until its end, when fruits are ripening, and the water level increases in the environment. It is however worth saying that in case of high availability of water either through rainfall or irrigation, the probability of germination of the conidia is high and reaches percentages up to 90% after 6 h as in the isolates COL‐1, COL‐4, and COL‐6.

Besides this general overview, our study highlighted the differences between the isolates as well: for instance, the slowest conidial germination and mycelial growth rates have been observed for the isolate COL‐3, setting it apart from the other isolates, which showed similar patterns. These differences may be due to the expression of some specific genes, as reported by (Li et al., 2021), responsible for producing specific proteins such as the Heterotrimeric G protein. This protein plays an important role in signal transduction in filamentous fungi, and the deletion the CgGa1 subunit negatively affects growth, asexual and sexual sporulation, appressorium formation, penetration, and pathogenicity of *C*. *gloeosporioides*.

If we look at the differences assessed on the mycelial growth, we can assert that except for the isolate COL‐3, all the others have almost the same behaviour within the optimal thermal range (between 20 and 30°C), or at least that the differences between the isolates is minimal. The higher variability that occurs going toward the lower and the upper thermal limits, is instead worthy of further investigation given the role that it may have on primary infections or the mycelium's survival during summer/winter.

*Colletotrichum fioriniae* is an important hemibiotrophic pathogen of the *C*. *acutatum* species complex that is present on different host plants. For instance, in South Korea, it is often reported on peach (Lee et al., 2018), in China it is recurrently present on *Vaccinium corymbosum* (Castro et al., 2023) and kiwifruit (Xu et al., 2023), and in the United States it is has been reported on grapevine (Nigar et al., 2023). The wide number of hosts and the increasing spread in different parts of the world demonstrates how *Colletotrichum* spp. can easily adapt to different plant species and disparate environmental conditions. This is confirmed in Italy where, besides olive trees, *C*. *fioriniae* was reported as causal agent of post‐harvest bitter rot of apple (Carneiro & Baric, 2021), of anthracnose on ornamental plants (Guarnaccia et al., 2021), walnut (Luongo et al., 2022) and beech (Giubilei et al., 2023). It is worth saying that before Italy, where it was first detected in Calabria and Lazio regions (Riolo & Cacciola, 2022), this pathogen has been found on olive cultivations in other areas worldwide, such as Australia (2009), Uruguay (2010), Portugal (2021), and California (2017; Moral et al., 2021).

The spread of this pathogen, as well as its adaptability, is endorsing control actions devoted to constraining the epidemics and development of accurate DSS based on mathematical models. Similar approaches have been carried out for several insect pests and pathogens (e.g., Donatelli et al., 2017; González‐Domínguez et al., 2014, 2020; Pfab et al., 2018; Rossi et al., 2003; Rossi, Caffi, Bugiani, et al., 2008; Rossi, Caffi, Giosuè, & Bugiani, 2008; Tonnang et al., 2017), but for each specific study case the estimation of the biological parameters was a fundamental preliminary step. Different authors already tried to develop models for different isolates or species of *Colletotrichum* (e.g., Perfect et al., 1999; Romero et al., 2022; Salotti et al., 2022, 2023) and our work is an additional piece of information for further comparisons and to understand, from a quantitative point of view, the differences in terms of environments.

## AUTHOR CONTRIBUTIONS

**Federico Brugneti:** Conceptualization (equal); data curation (equal); formal analysis (equal); investigation (equal); methodology (equal); validation (equal); visualization (equal); writing – original draft (lead); writing – review and editing (lead). **Luca Rossini:** Conceptualization (equal); data curation (equal); formal analysis (equal); investigation (equal); methodology (equal); software (equal); validation (equal); visualization (equal); writing – original draft (equal); writing – review and editing (equal). **Mounira Inas Drais:** Formal analysis (equal); investigation (equal); methodology (equal); validation (equal). **Silvia Turco:** Conceptualization (equal); formal analysis (equal); methodology (equal); supervision (equal); writing – original draft (equal); writing – review and editing (equal). **Angelo Mazzaglia:** Conceptualization (equal); funding acquisition (equal); project administration (equal); resources (equal); supervision (equal); writing – original draft (equal); writing – review and editing (equal).

## CONFLICT OF INTEREST STATEMENT

The authors declare no competing or financial interests.

## Supporting information


**Figure S1:** The phylogeny inferred by ML analysis is depicted in the figure.

**Table S1:** The sequences obtained were concatenated and compared with the corresponding sequences from *C*. *fioriniae* and related species from NCBI.

## Data Availability

The dataset and the scripts supporting the conclusion of this article are available in the GitHub repository, https://github.com/lucaros1190/Colletotrichum-Temperature.

## APPENDIX A

List of the primers used for the identification of the isolates considered in this study.

**Table:**

Primers	Sequence 5′‐3′	Amplicon length	Reference
ITS1 ITS4	TCCGTAGGTGAACCTGCGG TCCTCCGCTTATTGATATGC	~500 bp	White et al. (1990)
T1 Bt‐2b	AACATGCGTGAGATTGTAAGT ACCCTCAGTGTAGTGACCCTTGGC	~750 bp	O'Donnell and Cigelnik (1997) and Glass and Donaldson (1995)
ACT‐512F ACT‐783R	ATGTGCAAGGCCGGTTTCGC TACGAGTCCTTCTGGCCCAT	~226 bp	Carbone and Kohn (1999)
CHS‐79F CHS‐354R	TGGGGCAAGGATGCTTGGAAGAAG TGGAAGAACCATCTGTGAGAGTTG	~300 bp	Carbone and Kohn (1999)
CYLH3F CYLH3R	AGGTCCACTGGTGGCAAG AGCTGGATGTCCTTGGACTG	~412 bp	Crous et al. (2004)
GDF1 GDR1	GCCGTCAACGACCCCTTCATTGA GGGTGGAGTCGTACTTGAGCATGT	~200 bp	Guerber et al. (2003)

## References

[emi413275-bib-0001] Ballio, A. , Bottalico, A. , Buonocore, V. , Carilli, A. , Di Vittorio, V. & Graniti, A. (1969) Production and isolation of aspergillomarasmin B (lycomarasmic acid) from cultures of *Colletotrichum gloeosporioides* Penz. (*Gloeosporium olivarum* Aim.). *Phytopatologia Mediterranea* , 187–196.

[emi413275-bib-0002] Bates, D. , Mächler, M. , Bolker, B. & Walker, S. (2015) Fitting linear mixed‐effects models using *lme4* . Journal of Statistical Software, 67, 1–48.

[emi413275-bib-0003] Bellocchi, G. , Rivington, M. , Donatelli, M. & Matthews, K. (2011) Validation of biophysical models: issues and methodologies. In: Sustainable Agriculture, Vol. 2. Dordrecht: Springer Netherlands, pp. 577–603.

[emi413275-bib-0004] Briere, J.‐F. , Pracros, P. , Le Roux, A.‐Y. & Pierre, J.‐S. (1999) A novel rate model of temperature‐dependent development for arthropods. Environmental Entomology, 28, 22–29.

[emi413275-bib-0005] Cacciola, S.O. , Faedda, R. , Sinatra, F. , Agosteo, G.E. , Schena, L. & Frisullo, S. (2012) Olive antrachnose. Journal of Plant Pathology, 94, 29–44.

[emi413275-bib-0006] Capalbo, S.M. , Antle, J.M. & Seavert, C. (2017) Next generation data systems and knowledge products to support agricultural producers and science‐based policy decision making. Agricultural Systems, 155, 191–199. 28701812 10.1016/j.agsy.2016.10.009 PMC5485645

[emi413275-bib-0082] Carbone, I. & Kohn, L.M. (1999) A method for designing primer sets for speciation studies in filamentous ascomycetes. Mycologia, 91(3), 553–556.

[emi413275-bib-0007] Carneiro, G.A. & Baric, S. (2021) *Colletotrichum fioriniae* and *Colletotrichum godetiae* causing postharvest bitter rot of apple in South Tyrol (Northern Italy). Plant Disease, 105, 3118–3126. 33656363 10.1094/PDIS-11-20-2482-RE

[emi413275-bib-0008] Castro, J.F. , Millas, P. , Cisterna‐Oyarce, V. , Carrasco‐Fernández, J. , Santelices, C. , Muñoz, V. et al. (2023) First report of *Colletotrichum fioriniae* causing anthracnose fruit rot on *Vaccinium corymbosum* in Chile. Plant Disease, 107, 959.

[emi413275-bib-0009] Chen, Y. , Fu, D. , Wang, W. , Gleason, M.L. , Zhang, R. , Liang, X. et al. (2022) Diversity of *Colletotrichum* species causing apple bitter rot and glomerella leaf spot in China. Journal of Fungi, 8(7), 740. 35887495 10.3390/jof8070740 PMC9322356

[emi413275-bib-0084] Crous, P.W. , Groenewald, J.Z. , Risède, J.M. , Simoneau, P. & Hywel‐Jones, N.L. (2004) *Calonectria* species and their *Cylindrocladium* anamorphs: Species with sphaeropedunculate vesicles. Studies in Mycology, 50, 415–430. 10.3114/sim.55.1.213 PMC2104717 18490981

[emi413275-bib-0010] Damm, U. , Cannon, P.F. , Woudenberg, J.H.C. & Crous, P.W. (2012) The *Colletotrichum acutatum* species complex. Studies in Mycology, 73, 37–113. 23136458 10.3114/sim0010 PMC3458416

[emi413275-bib-0011] Donatelli, M. , Magarey, R.D. , Bregaglio, S. , Willocquet, L. , Whish, J.P.M. & Savary, S. (2017) Modelling the impacts of pests and diseases on agricultural systems. Agricultural Systems, 155, 213–224. 28701814 10.1016/j.agsy.2017.01.019 PMC5485649

[emi413275-bib-0012] Drais, M.I. , Pannucci, E. , Caracciolo, R. , Bernini, R. , Romani, A. , Santi, L. et al. (2021) Antifungal activity of hydroxytyrosol enriched extracts from olive mill waste against *Verticillium dahliae*, the cause of *Verticillium wilt* of olive. Phytopathologia Mediterranea, 60, 139–147.

[emi413275-bib-0013] Drais, M.I. , Rossini, L. , Turco, S. , Faluschi, A. & Mazzaglia, A. (2023) Modelling germination and mycelium growth rates of *Monostichella coryli* under constant temperature conditions. Fungal Ecology, 61, 101201.

[emi413275-bib-0014] Edgar, R.C. (2004) MUSCLE: multiple sequence alignment with high accuracy and high throughput. Nucleic Acids Research, 32, 1792–1797. 15034147 10.1093/nar/gkh340 PMC390337

[emi413275-bib-0015] Estrada, A.B. , Dodd, J.C. & Jeffries, P. (2000) Effect of humidity and temperature on conidial germination and appressorium development of two Philippine isolates of the mango anthracnose pathogen *Colletotrichum gloeosporioides* . Plant Pathology, 49, 608–618.

[emi413275-bib-0016] Faedda, R. , Agosteo, G.E. , Schena, L. , Mosca, S. , Frisullo, S. , Magnano Di San Lio, G. et al. (2011) *Colletotrichum Clavatum* sp. nov. Identified as the causal agent of olive anthracnose in Italy. Phytopathologia Mediterranea, 50(2), 283–302.

[emi413275-bib-0017] Fraga, H. , Moriondo, M. , Leolini, L. & Santos, J.A. (2020) Mediterranean olive orchards under climate change: a review of future impacts and adaptation strategies. Agronomy, 11, 56.

[emi413275-bib-0018] Gabriel y Galán, J.M. , Prada, C. , Martínez‐Calvo, C. & Lahoz‐Beltrá, R. (2015) A Gompertz regression model for fern spores germination. An Anales del Jardín Botánico de Madrid, 72, e015.

[emi413275-bib-0019] Garcia‐Lopez, M.T. , Serrano, M.S. , Camiletti, B.X. , Gordon, A. , Estudillo, C. , Trapero, A. et al. (2023) Study of the competition between *Colletotrichum godetiae* and *C*. *Nymphaeae*, two pathogenic species in olive. Scientific Reports, 13, 5344. 37005485 10.1038/s41598-023-32585-6 PMC10067957

[emi413275-bib-0080] Glass, N.L. & Donaldson, G.C. (1995) Development of primer sets designed for use with the PCR to amplify conserved genes from filamentous ascomycetes. Applied and Environmental Microbiology, 61(4), 1323–1330. 7747954 10.1128/aem.61.4.1323-1330.1995 PMC167388

[emi413275-bib-0020] Giubilei, I. , Brugneti, F. , Turco, S. , Drais, M.I. & Mazzaglia, A. (2023) First report of anthracnose on *Fagus sylvatica* caused by *Colletotrichum fioriniae* in Italy. New Disease Reports, 48, e12226.

[emi413275-bib-0021] González‐Domínguez, E. , Armengol, J. & Rossi, V. (2014) Development and validation of a weather‐based model for predicting infection of loquat fruit by *Fusicladium eriobotryae* . PLoS One, 9, e107547. 25233340 10.1371/journal.pone.0107547 PMC4169414

[emi413275-bib-0022] González‐Domínguez, E. , Fedele, G. , Salinari, F. & Rossi, V. (2020) A general model for the effect of crop management on plant disease epidemics at different scales of complexity. Agronomy, 10, 462.

[emi413275-bib-0023] Gougouli, M. & Koutsoumanis, K.P. (2012) Modeling germination of fungal spores at constant and fluctuating temperature conditions. International Journal of Food Microbiology, 152, 153–161. 21885146 10.1016/j.ijfoodmicro.2011.07.030

[emi413275-bib-0024] Guarnaccia, V. , Martino, I. , Gilardi, G. , Garibaldi, A. & Gullino, M.L. (2021) *Colletotrichum* spp. causing anthracnose on ornamental plants in northern Italy. Journal of Plant Pathology, 103, 127–137.

[emi413275-bib-0081] Guerber, J.C. , Liu, B. , Correll, J.C. & Johnston, P.R. (2003) Characterization of diversity in *Colletotrichum acutatum* sensu lato by sequence analysis of two gene introns, mtDNA and intron RFLPs, and mating compatibility. Mycologia, 95(5), 872–895. 21148995

[emi413275-bib-0025] Hilário, S. , Santos, L. & Alves, A. (2021) *Diaporthe amygdali*, a species complex or a complex species? Fungal Biology, 125, 505–518. 34140147 10.1016/j.funbio.2021.01.006

[emi413275-bib-0026] Hothorn, T. , Bretz, F. & Westfall, P. (2008) Simultaneous inference in general parametric models. Biometrical Journal, 50, 346–363. 18481363 10.1002/bimj.200810425

[emi413275-bib-0027] Ikemoto, T. & Kiritani, K. (2019) Novel method of specifying low and high threshold temperatures using thermodynamic SSI model of insect development. Environmental Entomology, 48, 479–488. 30993314 10.1093/ee/nvz031

[emi413275-bib-0028] Jin, J. , Quinn, B.K. & Shi, P. (2022) The modified Brière equation and its applications. Plants, 11, 1769. 35807720 10.3390/plants11131769 PMC9269267

[emi413275-bib-0029] Knight, J.D. & Mumford, J.D. (1994) Decision support systems in crop protection. Outlook on Agriculture, 23, 281–285.

[emi413275-bib-0030] Körner, O. , Holst, N. & De Visser, P. (2014) A model‐based decision support tool for grey mould prediction. Acta Horticulturae, 1037, 569–574.

[emi413275-bib-0031] Lee, D. , Hassan, O. , Kim, C. & Chang, T. (2018) First report of peach (*Prunus persica*) anthracnose caused by *Colletotrichum fioriniae* in Korea. Plant Disease, 102, 2650.

[emi413275-bib-0032] Li, X. , Ke, Z. , Xu, S. , Tang, W. & Liu, Z. (2021) The G‐protein alpha subunit CgGa1 mediates growth, sporulation, penetration and pathogenicity in *Colletotrichum gloeosporioides* . Microbial Pathogenesis, 161, 105254. 34687840 10.1016/j.micpath.2021.105254

[emi413275-bib-0033] Liu, F. , Wang, M. , Damm, U. , Crous, P.W. & Cai, L. (2016) Species boundaries in plant pathogenic fungi: a *Colletotrichum* case study. BMC Evolutionary Biology, 16, 1–14. 27080690 10.1186/s12862-016-0649-5 PMC4832473

[emi413275-bib-0034] Luongo, L. , Galli, M. , Garaguso, I. , Petrucci, M. & Vitale, S. (2022) First report of *Colletotrichum fioriniae* and *C*. *nymphaeae* as causal agents of anthracnose on walnut in Italy. Plant Disease, 106, 327.34372683

[emi413275-bib-0035] Marcelino, J. , Giordano, R. , Gouli, S. , Gouli, V. , Parker, B.L. , Skinner, M. et al. (2008) *Colletotrichum acutatum* var. *fioriniae* (teleomorph: *Glomerella acutata* var. *fioriniae* var. nov.) infection of a scale insect. Mycologia, 100(3), 353–374. 18751543 10.3852/07-174r

[emi413275-bib-0036] Moral, J. , Agustí‐Brisach, C. , Raya, M.C. , Jurado‐Bello, J. , López‐Moral, A. , Roca, L.F. et al. (2021) Diversity of *Colletotrichum* species associated with olive anthracnose worldwide. Journal of Fungi, 7, 741. 34575779 10.3390/jof7090741 PMC8466006

[emi413275-bib-0037] Moral, J. , Muñoz‐Díez, C. , González, N. , Trapero, A. & Michailides, T.J. (2010) Characterization and pathogenicity of *Botryosphaeriaceae* species collected from olive and other hosts in Spain and California. Phytopathology, 100, 1340–1351. 20731532 10.1094/PHYTO-12-09-0343

[emi413275-bib-0038] Moral, J. & Trapero, A. (2012) Mummified fruit as a source of inoculum and disease dynamics of olive anthracnose caused by *Colletotrichum* spp. Phytopathology, 102, 982–989. 22957822 10.1094/PHYTO-12-11-0344

[emi413275-bib-0039] Mosca, S. , Li Destri Nicosia, M.G. , Cacciola, S.O. & Schena, L. (2014) Molecular analysis of *Colletotrichum* species in the carposphere and phyllosphere of olive. PLoS One, 9, e114031. 25501572 10.1371/journal.pone.0114031 PMC4263604

[emi413275-bib-0040] Mousavi, S. , De La Rosa, R. , Moukhli, A. , El Riachy, M. , Mariotti, R. , Torres, M. et al. (2019) Plasticity of fruit and oil traits in olive among different environments. Scientific Reports, 9, 16968. 31740728 10.1038/s41598-019-53169-3 PMC6861299

[emi413275-bib-0041] Nigar, Q. , Cadle‐Davidson, L. , Gadoury, D.M. & Hassan, M.U. (2023) First report of *Colletotrichum fioriniae* causing grapevine anthracnose in New York. Plant Disease, 107, 223.

[emi413275-bib-0079] O'Donnell, K. & Cigelnik, E. (1997) Two divergent intragenomic rDNA ITS2 types within a monophyletic lineage of the fungusfusariumare nonorthologous. Molecular Phylogenetics and Evolution, 7(1), 103–116. 9007025 10.1006/mpev.1996.0376

[emi413275-bib-0042] Omuse, E.R. , Niassy, S. , Wagacha, J.M. , Ong'amo, G.O. , Azrag, A.G.A. & Dubois, T. (2021) Suitable models to describe the effect of temperature on conidial germination and mycelial growth of *Metarhizium anisopliae* and *Beauveria bassiana* . Biocontrol Science and Technology, 32(3), 281–298.

[emi413275-bib-0043] Perfect, S.E. , Hughes, H.B. , O'Connell, R.J. & Green, J.R. (1999) *Colletotrichum*: a model genus for studies on pathology and fungal–plant interactions. Fungal Genetics and Biology, 27, 186–198. 10441444 10.1006/fgbi.1999.1143

[emi413275-bib-0044] Peterson, R.A. (2021) Finding optimal normalizing transformations via *bestNormalize* . R Journal, 13, 310–329.

[emi413275-bib-0045] Peterson, R.A. & Cavanaugh, J.E. (2020) Ordered quantile normalization: a semiparametric transformation built for the cross‐validation era. Journal of Applied Statistics, 47, 2312–2327. 35707424 10.1080/02664763.2019.1630372 PMC9042069

[emi413275-bib-0046] Pfab, F. , Stacconi, M.V.R. , Anfora, G. , Grassi, A. , Walton, V. & Pugliese, A. (2018) Optimized timing of parasitoid release: a mathematical model for biological control of *Drosophila suzukii* . Theoretical Ecology, 11, 489–501.

[emi413275-bib-0047] Prosser, J.I. (1995) Mathematical modelling of fungal growth. In: The growing fungus. Dordrecht: Springer Netherlands, pp. 319–335.

[emi413275-bib-0048] R Core Team . (2018) R: a language and environment for statistical computing. Vienna, Austria: R Foundation for Statistical Computing.

[emi413275-bib-0049] Riolo, M. & Cacciola, S.O. (2022) First report of *Colletotrichum fioriniae* associated with olive anthracnose in Italy. Journal of Plant Pathology, 105, 363.

[emi413275-bib-0050] Robinet, C. , Kehlenbeck, H. , Kriticos, D.J. , Baker, R.H.A. , Battisti, A. , Brunel, S. et al. (2012) A suite of models to support the quantitative assessment of spread in Pest risk analysis. PLoS One, 7, e43366. 23056174 10.1371/journal.pone.0043366 PMC3467266

[emi413275-bib-0051] Romero, J. , Santa‐Bárbara, A.E. , Moral, J. , Agustí‐Brisach, C. , Roca, L.F. & Trapero, A. (2022) Effect of latent and symptomatic infections by *Colletotrichum godetiae* on oil quality. European Journal of Plant Pathology, 163, 545–556.

[emi413275-bib-0052] Rossi, V. , Caffi, T. , Bugiani, R. , Spanna, F. & Valle, D.D. (2008) Estimating the germination dynamics of *Plasmopara viticola* oospores using hydro‐thermal time. Plant Pathology, 57, 216–226.

[emi413275-bib-0053] Rossi, V. , Caffi, T. , Giosuè, S. & Bugiani, R. (2008) A mechanistic model simulating primary infections of downy mildew in grapevine. Ecological Modelling, 212, 480–491.

[emi413275-bib-0054] Rossi, V. , Giosuè, S. , Pattori, E. , Spanna, F. & Del Vecchio, A. (2003) A model estimating the risk of fusarium head blight on wheat. EPPO Bulletin, 33, 421–425.

[emi413275-bib-0055] Rossi, V. , Sperandio, G. , Caffi, T. , Simonetto, A. & Gilioli, G. (2019) Critical success factors for the adoption of decision tools in IPM. Agronomy, 9, 710.

[emi413275-bib-0056] Rossini, L. , Bono Rosselló, N. , Contarini, M. , Speranza, S. & Garone, E. (2022) Modelling ectotherms' populations considering physiological age structure and spatial motion: a novel approach. Ecological Informatics, 70, 101703.

[emi413275-bib-0057] Rossini, L. , Bono Rosselló, N. , Speranza, S. & Garone, E. (2021) A general ODE‐based model to describe the physiological age structure of ectotherms: description and application to *Drosophila suzukii* . Ecological Modelling, 456, 109673.

[emi413275-bib-0058] Rossini, L. , Bruzzone, O.A. , Contarini, M. , Bufacchi, L. & Speranza, S. (2022) A physiologically based ODE model for an old pest: modeling life cycle and population dynamics of *Bactrocera oleae* (Rossi). Agronomy, 12, 2298.

[emi413275-bib-0059] Rossini, L. , Contarini, M. , Severini, M. & Speranza, S. (2020) Reformulation of the distributed delay model to describe insect pest populations using count variables. Ecological Modelling, 436, 109286.

[emi413275-bib-0060] Rossini, L. , Severini, M. , Contarini, M. & Speranza, S. (2019a) A novel modelling approach to describe an insect life cycle vis‐à‐vis plant protection: description and application in the case study of *Tuta absoluta* . Ecological Modelling, 409, 108778.

[emi413275-bib-0061] Rossini, L. , Severini, M. , Contarini, M. & Speranza, S. (2019b) Use of ROOT to build a software optimized for parameter estimation and simulations with distributed delay model. Ecological Informatics, 50, 184–190.

[emi413275-bib-0062] Rossini, L. , Severini, M. , Contarini, M. & Speranza, S. (2020) *EntoSim*, a ROOT‐based simulator to forecast insects' life cycle: description and application in the case of *Lobesia botrana* . Crop Protection, 129, 105024.

[emi413275-bib-0063] Rossini, L. , Speranza, S. & Contarini, M. (2020) Distributed delay model and Von Foerster's equation: different points of view to describe insects' life cycles with chronological age and physiological time. Ecological Informatics, 59, 101117.

[emi413275-bib-0064] Rossini, L. , Virla, E.G. , Albarracín, E.L. , Van Nieuwenhove, G.A. & Speranza, S. (2021) Evaluation of a physiologically based model to predict *Dalbulus maidis* occurrence in maize crops: validation in two different subtropical areas of South America. Entomologia Experimentalis et Applicata, 169, 597–609.

[emi413275-bib-0065] Rosso, L. , Lobry, J.R. , Bajard, S. & Flandrois, J.P. (1995) Convenient model to describe the combined effects of temperature and pH on microbial growth. Applied and Environmental Microbiology, 61, 610–616. 16534932 10.1128/aem.61.2.610-616.1995 PMC1388350

[emi413275-bib-0066] Rosso, L. , Lobry, J.R. & Flandrois, J.P. (1993) An unexpected correlation between cardinal temperatures of microbial growth highlighted by a new model. Journal of Theoretical Biology, 162, 447–463. 8412234 10.1006/jtbi.1993.1099

[emi413275-bib-0067] Salotti, I. , Ji, T. & Rossi, V. (2022) Temperature requirements of *Colletotrichum* spp. belonging to different clades. Frontiers in Plant Science, 13, 953760. 35937340 10.3389/fpls.2022.953760 PMC9354546

[emi413275-bib-0068] Salotti, I. , Liang, Y.‐J. , Ji, T. & Rossi, V. (2023) Development of a model for *Colletotrichum* diseases with calibration for phylogenetic clades on different host plants. Frontiers in Plant Science, 14, 1069092. 37063197 10.3389/fpls.2023.1069092 PMC10090521

[emi413275-bib-0069] Sarkar, D. (2008) Lattice: multivariate data visualization with R. New York: Springer.

[emi413275-bib-0070] Schena, L. , Abdelfattah, A. , Mosca, S. , Li Destri Nicosia, M.G. , Agosteo, G.E. & Cacciola, S.O. (2017) Quantitative detection of *Colletotrichum godetiae* and *C*. *acutatum sensu stricto* in the phyllosphere and carposphere of olive during four phenological phases. European Journal of Plant Pathology, 149, 337–347.

[emi413275-bib-0071] Searle, S.R. , Speed, F.M. & Milliken, G.A. (1980) Population marginal means in the linear model: an alternative to least squares means. The American Statistician, 34, 216–221.

[emi413275-bib-0072] Shivas, R.G. & Tan, Y.P. (2009) A taxonomic re‐assessment of *Colletotrichum acutatum*, introducing *C*. *fioriniae* comb. et stat. nov. and *C*. *simmondsii* sp. nov. Fungal Diversity, 39, 111–122.

[emi413275-bib-0073] Stamatakis, A. (2014) RAxML version 8: a tool for phylogenetic analysis and post‐analysis of large phylogenies. Bioinformatics, 30, 1312–1313. 24451623 10.1093/bioinformatics/btu033 PMC3998144

[emi413275-bib-0074] Talhinhas, P. & Baroncelli, R. (2021) *Colletotrichum* species and complexes: geographic distribution, host range and conservation status. Fungal Diversity, 110, 109–198.

[emi413275-bib-0075] Talhinhas, P. , Mota‐Capitão, C. , Martins, S. , Ramos, A.P. , Neves‐Martins, J. , Guerra‐Guimarães, L. et al. (2011) Epidemiology, histopathology and aetiology of olive anthracnose caused by *Colletotrichum acutatum* and *C*. *gloeosporioides* in Portugal. Plant Pathology, 60, 483–495.

[emi413275-bib-0076] Tonnang, H.E.Z. , Hervé, B.D.B. , Biber‐Freudenberger, L. , Salifu, D. , Subramanian, S. , Ngowi, V.B. et al. (2017) Advances in crop insect modelling methods – towards a whole system approach. Ecological Modelling, 354, 88–103.

[emi413275-bib-0083] White, T.J. , Bruns, T. , Lee, S.J.W.T. & Taylor, J. (1990) Amplification and direct sequencing of fungal ribosomal RNA genes for phylogenetics. PCR Protocols: A Guide to Methods and Applications, 18(1), 315–322.

[emi413275-bib-0077] Xu, X. , Zheng, D. , Lan, J. , Song, W. , Song, S. , Huang, L. et al. (2023) First report of postharvest anthracnose of kiwifruit caused by *Colletotrichum fioriniae* in Liaoning and Sichuan province, China. Plant Disease, 107, 1236.

[emi413275-bib-0078] Zhang, Q. , Nizamani, M.M. , Feng, Y. , Yang, Y.Q. , Jayawardena, R.S. , Hyde, K.D. et al. (2023a) Genome‐scale and multi‐gene phylogenetic analyses of *Colletotrichum* spp. host preference and associated with medicinal plants. Mycosphere, 14(2), 1–106.

[emi413275-bib-0085] Zhang, Y. , Chen, J. , Manawasinghe, I. , Lin, Y. , Jayawardena, R. , McKenzie, E. et al. (2023b) Identification and characterization of colletotrichum species associated with ornamental plants in Southern China. Mycosphere, 14(si2), 262–302. Available from: 10.5943/mycosphere/14/si2/5

