# Supplementary figures and images for: Effect of temperature on in vitro germination and growth of Colletotrichum fioriniae, a new emerging pathogen of olive fruits
Source: Environ Microbiol Rep. 2024 Sep 4;16(5):e13275. doi: 10.1111/1758-2229.13275 (PMC11372289; doi:10.1111/1758-2229.13275)

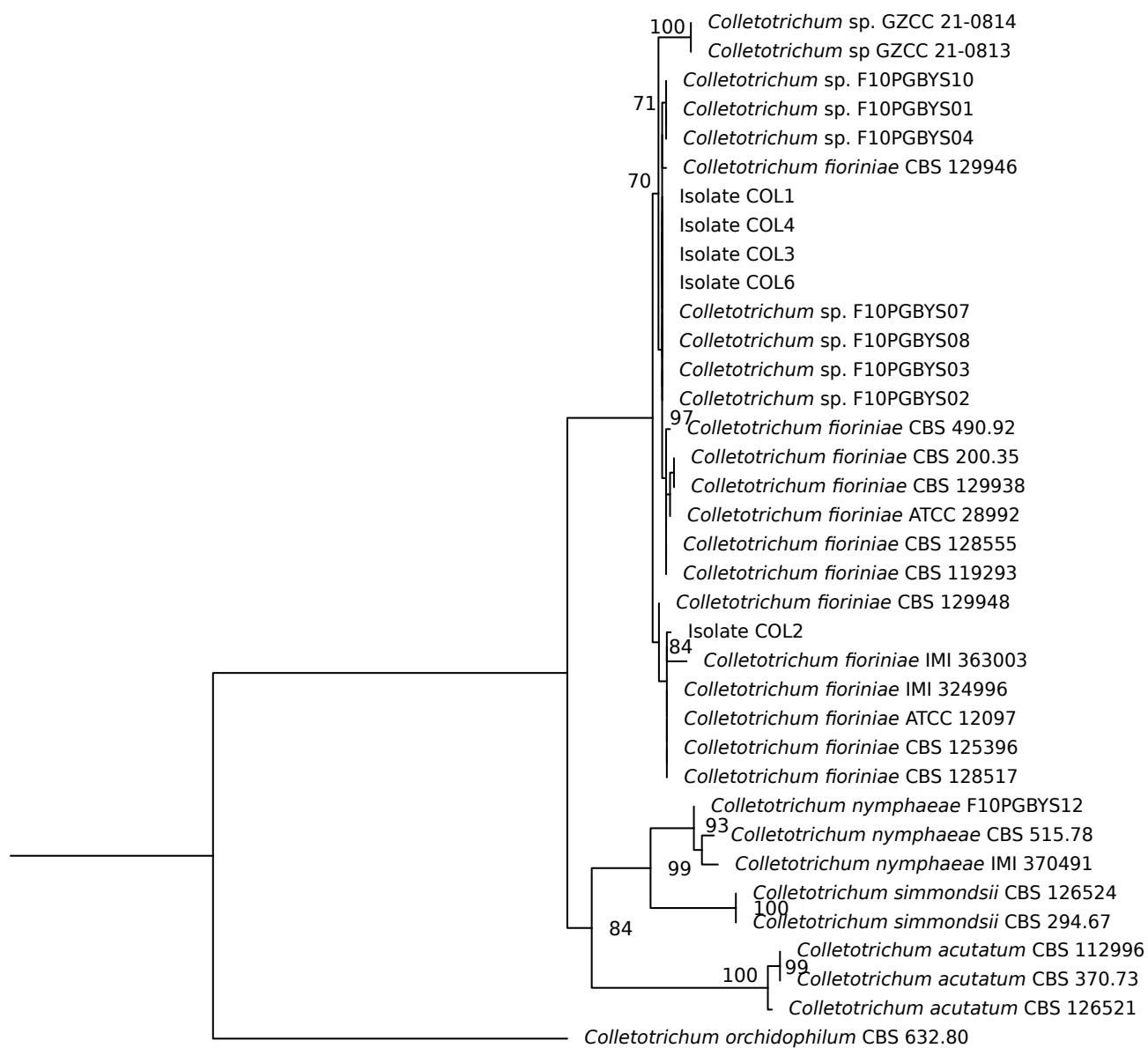

0.009

Supplement: Supplementary file 1 — Figure S1: The phylogeny inferred by ML analysis is depicted in the figure. [file EMI4-16-e13275-s002.pdf]
